# Integrated Analysis of Drug-Induced Gene Expression Profiles Predicts Novel hERG Inhibitors

**DOI:** 10.1371/journal.pone.0069513

**Published:** 2013-07-23

**Authors:** Joseph J. Babcock, Fang Du, Kaiping Xu, Sarah J. Wheelan, Min Li

**Affiliations:** 1 The Solomon H. Snyder Department of Neuroscience and High Throughput Biology Center, The Johns Hopkins University School of Medicine, Baltimore, Maryland, United States of America; 2 Johns Hopkins Ion Channel Center (JHICC), The Johns Hopkins University School of Medicine, Baltimore, Maryland, United States of America; 3 Department of Oncology, Division of Biostatistics and Bioinformatics, The Johns Hopkins University School of Medicine, Baltimore, Maryland, United States of America; University of California, Irvine, United States of America

## Abstract

Growing evidence suggests that drugs interact with diverse molecular targets mediating both therapeutic and toxic effects. Prediction of these complex interactions from chemical structures alone remains challenging, as compounds with different structures may possess similar toxicity profiles. In contrast, predictions based on systems-level measurements of drug effect may reveal pharmacologic similarities not evident from structure or known therapeutic indications. Here we utilized drug-induced transcriptional responses in the Connectivity Map (CMap) to discover such similarities among diverse antagonists of the human *ether-à-go-go* related (hERG) potassium channel, a common target of promiscuous inhibition by small molecules. Analysis of transcriptional profiles generated in three independent cell lines revealed clusters enriched for hERG inhibitors annotated using a database of experimental measurements (hERGcentral) and clinical indications. As a validation, we experimentally identified novel hERG inhibitors among the unannotated drugs in these enriched clusters, suggesting transcriptional responses may serve as predictive surrogates of cardiotoxicity complementing existing functional assays.

## Introduction

While the single-target approach to drug discovery seeks “silver bullets” that selectively modulate disease-related proteins, recent work has emphasized the often promiscuous interactions of both marketed and candidate therapeutics [1–3]. The positive impact of such polypharmacology includes the potential to discover novel clinical uses for previously approved medications [4–6]. However, it also suggests that drugs may share similar and undesirable side effects despite unrelated chemical structures or primary mechanisms-of-action (MOA). While existing quantitative structure activity relationship (QSAR) methods have leveraged structural features of small molecules to predict toxicity, the difficulty of applying such techniques to chemicals that vary substantially from the model inputs has been described, particularly in cases where toxicity is linked to the metabolic by-products of a compound [7,8]. Thus alternative descriptors, such as measurements of drug effects that probe the complex physiology of the cell, may potentially reveal commonalities aiding the prediction of toxicity independent of chemical structure as represented, for example, by conventional chemical fingerprints. Here, we explored similarities in drug-induced transcriptional effects using the Connectivity Map (CMap), a collection of Affymetrix™ microarray profiles generated by treating three independent lineages of cancer cell lines with small molecule drugs [9]. In previous applications, analysis of the CMap has associated transcriptional signatures to known MOAs or disease states, allowing the discovery of novel modulators of autophagy, small cell lung cancer proliferation, and inflammatory bowel disease [5,6,10]. Similarly, computational studies have identified correlations between known drug side effects and transcriptional responses in the CMap [11,12]. Thus, we hypothesized that this data might also be used to predict and verify novel toxicities, which we demonstrate by integrating the CMap with experimentally measured inhibition data for the human *ether-à-go-go* related (hERG) potassium channel and literature annotations to identify novel antagonists of this important anti-target of many drugs.

Promiscuous inhibition of the hERG channel by therapeutically and structurally diverse drugs prolongs the QT interval quantified by surface electrocardiogram (ECG) [13]. This phenomenon, known as drug-induced Long QT (LQT) syndrome, is a risk factor for sudden cardiac death [13]. To date, the lack of universal chemical patterns and diversity of primary clinical targets among known hERG inhibitors have impeded effective risk assessment of this side effect using computational methods, and experimental evaluation using the “gold standard” of electrophysiology remains an important step in therapeutic development. Such electrophysiological recordings, utilizing recombinantly expressed hERG channels [14–16] as well as patient-derived cardiomyocytes [17,18], have afforded valuable experimental opportunities to study the potential LQT side effects of small molecules. More recently, the development of high-throughput electrophysiology platforms has facilitated systematic evaluation of hERG inhibition in large compound collections [19,20]. Concurrently, potential global physiological readouts for channel function are suggested by behavioral assays in model organisms such as *C. elegans* and *D. rerio* [21,22], as well as reports linking channel activity to tumor migration and volume [23,24], indicating these phenomena may conceivably be used as ways to probe hERG liability. Computationally, hERG inhibition has also been correlated with the proximity of a drug’s therapeutic target to hERG in a protein–protein interaction network [25].

Our present analysis integrates earlier results in which we have independently profiled over 300,000 compounds (including approximately half of the CMap compounds) in the NIH Molecular Library Small Molecule Repository (MLSMR) for their ability to inhibit hERG current in a high-throughput electrophysiological assay [26]. Combining our database with additional publicly available annotations for LQT side effect allowed us to identify clusters of drugs with similar expression profiles in the CMap enriched for channel inhibitors. Drugs of unknown hERG liability within these clusters, through the principle of ‘guilt by association’, were then experimentally validated using an electrophysiology assay. These results advance the hypothesis that structurally diverse hERG inhibitors mediate similar physiological effects revealed by transcriptional response profiles, even in cell lines not derived from a cardiac lineage and potentially independent of hERG expression. Thus, gene expression signatures may serve as a proxy measurement correlated with reduction of hERG current, and find practical application as a high-throughput platform to complement existing electrophysiological assays. More generally, these analyses suggest that side effect profiles as well as primary MOAs may be predictively correlated with similarities in drug-induced gene expression responses independent of chemical structure.

## Results

### Microarray normalization and analysis

**Figure 1:**
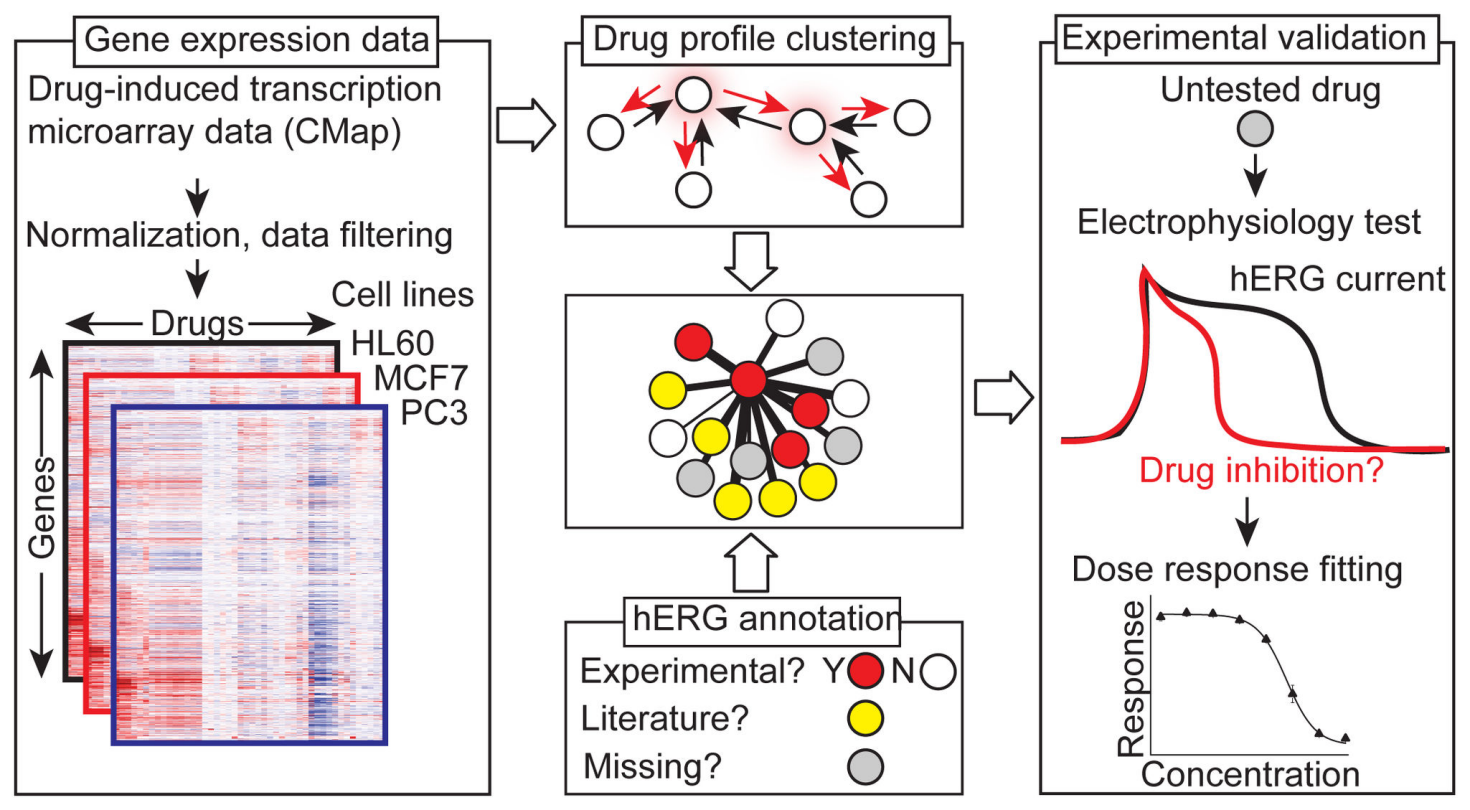
Pipeline for construction and analysis of drug transcriptional response network. Raw microarray data for drugs profiled in three cancer cell lines in the Connectivity Map (left) are normalized and clustered using affinity propagation (top center) based on similarities in drug-induced gene expression profiles (nodes) to yield clusters with a characteristic “exemplar” (highlighted by red) representing the expression profile shared by cluster members. The resulting clusters (middle center) are annotated for experimental and clinical evidence of hERG inhibition (bottom center), and enrichment analysis conducted to find clusters with a statistically significant fraction of hERG inhibitors. Unannotated compounds in these enriched clusters (top right) are then experimentally assessed for hERG inhibition in a high-throughput electrophysiology assay (middle right) to yield potency values (bottom right).

Our analysis pipeline is outlined in Figure **1**. The Connectivity Map (CMap) (http://www.broadinstitute.org/cmap/) [9] is a collection of Affymetrix™ microarray gene expression profiles representing the responses of three cancer cell lines (breast cancer: MCF7, prostate cancer: PC3, and leukemia: HL60) to small molecule treatments in comparison to dimethyl sulfoxide (DMSO) treated samples used as vehicle-treatment controls for these studies because most drugs are dissolved using DMSO as a solvent. The full dataset currently contains one or more measurements for 1,309 unique substances at varying concentrations. To begin our investigation, we normalized the downloaded CMap data and corrected for batch effects (similarity among arrays correlated with the experimental group in which they were processed rather than biological annotations) by centering the mean expression value per probeset in each batch at zero, as previously proposed [27]. Thus, the resulting profiles represent changes in gene expression dependent on drug effect rather than experimental batch. Following this correction, we also noted a sub-population of arrays representing replicate treatments (the same drug tested against the same cell line at the same concentration) that have approximately zero Pearson correlation (as judged by the correlation of log_2_ fold changes versus vehicle control). By using thresholds for minimum log_2_ fold gene expression change among probesets on an array, it is possible to split this population into two groups of correlated and uncorrelated replicates (Figure **S1**). We propose that these uncorrelated replicates may represent “transcriptionally silent” drugs where the observed response is random signal variation and thus not preserved between repeats, and thus removed them from our sample prior to downstream analysis. Finally, we used the batch-corrected DMSO-treated samples to identify probesets in each drug-induced expression profile whose fold change lies outside the range of variation exhibited by the same probeset in DMSO-treated control samples measured in the same cell background. Table **1** summarizes the number of arrays filtered and unique drug instances filtered during each stage of this analysis, as well as the number of experimental and annotated hERG inhibitors present in each of these samples. The final sample of 673 unique drugs contains 1,033 drug-cell line combinations (‘Merge Replicates’ row in Table 1), as some drugs were profiled in more than one cell background, with 62 experimentally and 57 clinically annotated as hERG inhibitors.

**Table 1:** Microarray data processing statistics.

Dataset	Arrays (Drugs)	Unique Drugs	Exp. Blockers (Unique)		LQT Drugs (Unique)	
CMap Build 02	7,056 (6,100)	1,309	324 (61)		284 (54)	
HT-HG-U133A	6,029 (5,242)	1,219	271 (60)		234 (52)	
Batch Correction	5,454 (4,754)	1,145	253 (60)		222 (52)	
Non-Silent Drugs	2,119 (1,419)	673	88 (37)		76 (35)	
Merge Replicates	-	673	62 (37)		57 (35)	

For each step of data processing (CMap Build 02 = full dataset, HT-HG-U133A = platform sub-selection, Batch Correction = arrays from batches of size > 25, mode test concentration for a given drug, Non-Silent Drugs = drugs passing ‘silent’ transcription filters, Merge Replicates = average of arrays from the same drug and cell background), the number of unique drugs, experimentally determined hERG blockers (Exp. Blockers) with IC_50_ < 10 µM (parenthesis unique experimentally determined blockers) and drugs (LQT Drugs) from LQT drugs lists not present in the experimental blockers (parenthesis annotated LQT drugs) are listed.

### Enrichment of structurally diverse hERG inhibitors through transcriptional response similarity

Following pre-processing, we clustered the resulting collection of drug-induced gene expression profiles using affinity propagation [28], an unsupervised learning algorithm that automatically identifies the optimal number of clusters in a dataset using an input of all pairwise similarities (here, the pairwise Pearson correlations between expression profiles). Each cluster generated by this procedure contains an “exemplar”, a single member that best characterizes the pattern shared by the members of the group. To identify higher-level relationships between individual clusters, we further aggregated the data by recursively re-clustering these exemplars to attain a global view of the number of characteristic patterns of drug-induced gene expression changes in this collection. We integrated the gene expression measurements with annotations for hERG inhibition derived from two sources: a previously described dataset of electrophysiology measurements of hERG inhibition (http://www.hERGcentral.org) [26], and lists of drugs that have been clinically linked to LQT side effects (http://ww.sads.org.uk and http://www.qtdrugs.org). Drugs with records in hERGcentral were annotated as inhibitors if they decreased hERG current by 50% or more at 10 µM concentration, representing an IC_50_ value of approximately 10 µM or less. We selected this threshold as the dataset in hERGCentral contains inhibition measurements at 1 µM and 10 µM, and literature data is frequently annotated with potency (IC_50_) values, making these two concentrations the most convenient thresholds. We chose a less conservative (10 µM) threshold, as this value correctly identifies 40/53 (76%) of torsades de pointes (TdP)-risk drugs described in a literature survey [29]. The agreement between the hERGcentral measurements (continuous values) and existing LQT drug annotations (binary classifications) is demonstrated by a Wilcoxon rank-sum test comparing the median experimentally determined hERG inhibition values from hERGcentral for drugs with or without previous annotation for LQT side effects (p-value 5.7x10^-12^, Figure **S2**). Complete experimental and clinical annotations based on the criteria described above are given in Table **S1**.

**Figure 2:**
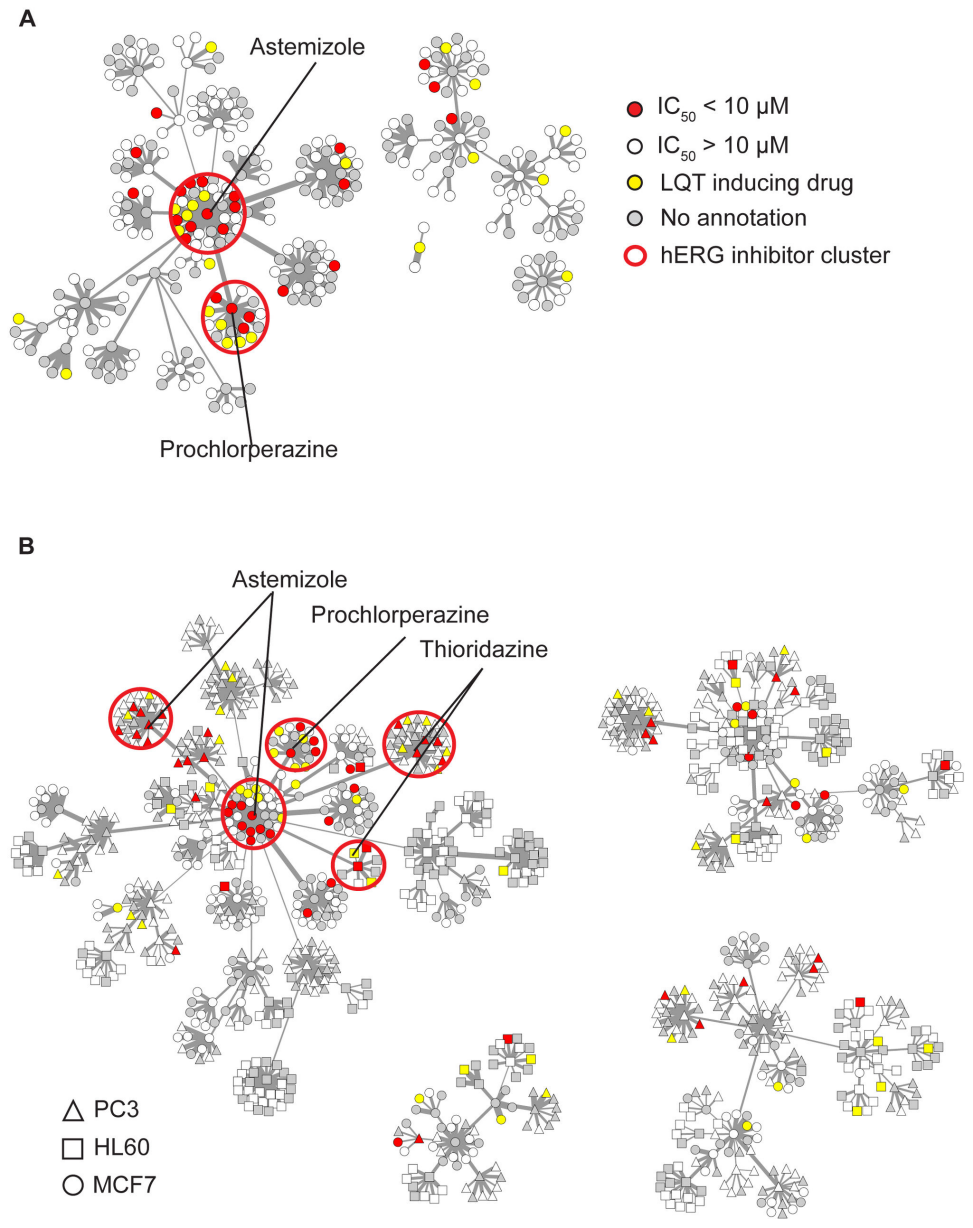
Network analysis of drug-induced gene expression profiles. (**A**) Drug-induced gene expression profiles tested in MCF7 (breast cancer) cells (nodes) are linked by shared expression patterns to a cluster exemplar (line width proportional to Pearson correlation) representing their characteristic response. Clusters enriched for literature or experimentally annotated hERG inhibitors are outlined in red. (**B**) Drug induced gene expression profiles generated from MCF7, PC3 (prostate cancer), and HL60 (leukemia) cell lines are clustered as in (**A**), with cell of origin indicated by node shape.

The clusters generated from the drug-induced gene expression profiles derived from the breast cancer cell line MCF7 are displayed in a network diagram in Figure **2A**, where nodes represent individual drugs and edge weights represent the magnitude of similarity (Pearson correlation coefficient) between a given drug’s expression profile and the exemplar of its cluster. We found that 2 of the 31 resulting clusters contained an enriched fraction of hERG inhibitors compared to randomized clusters of the same size (using a false discovery rate threshold of 0.2). Even after correction for experimental batch effects described above, clustering of all drug-induced expression profiles demonstrates assortment by cell background, suggesting the existence of cell line-specific drug effects (Figure 2B). However, as the exemplars of these sub-clusters are hierarchically merged, connections emerge between hERG inhibitor-enriched clusters derived from all three different cells lines, indicating the presence of general as well as cell background-specific responses (Figure 2B). This interpretation is supported by a scatterplot of average expression changes in these five hERG inhibitor-enriched clusters in the three cell lines, which indicates that some differentially expressed (DE) genes are shared (Figure **S3A**), as well as Venn diagrams indicating the overlap of DE genes between these sets (Figure **S3, B & C**). The clustering results for each of the networks in Figure **2** are given in Table **S2**.

**Figure 3:**
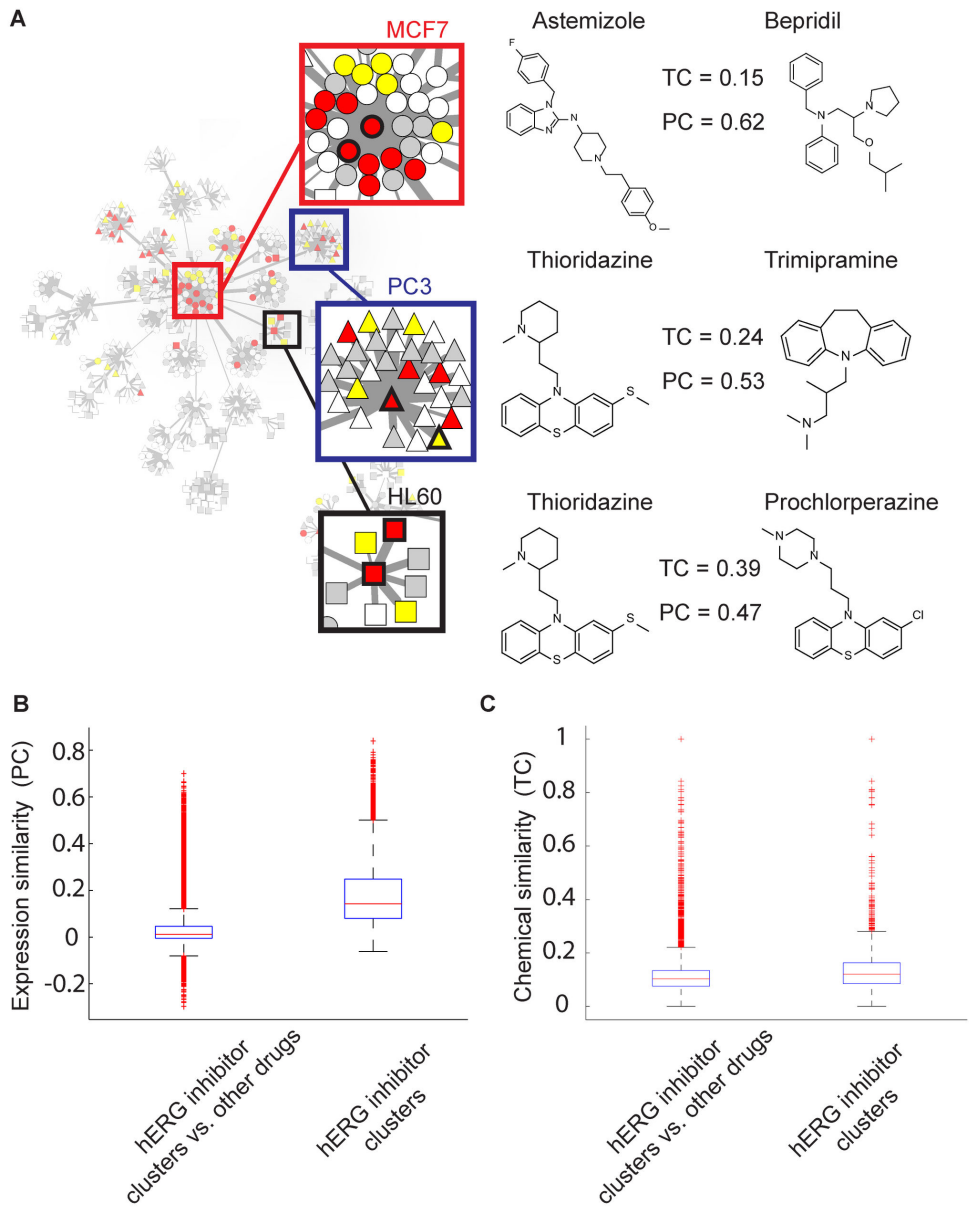
Expression and structural similarity of hERG inhibitor-enriched clusters. (**A**) Chemical similarity (Tanimoto coefficient = TC) computed from FCFP_6 circular fingerprints versus expression similarity (Pearson coefficient = PC) computed from drug-induced transcriptional response for selected hERG inhibitor-enriched clusters for MCF7 (top) PC3 (middle) and HL60 (bottom). Cluster in drug expression networks are highlighted, with example compounds outlined in black in inset (left column). Chemical structures are illustrated with corresponding chemical and expression similarity values. (**B**) Distribution of pairwise expression response similarities within hERG inhibitor-enriched clusters and between drugs in enriched and non-enriched clusters from Figure **2B**. (**C**) As (**B**), comparing distribution of chemical similarities.

Further, we note that many of the exemplars of these hERG inhibitor-enriched clusters are preserved between cell lines (Figure 2B). The enrichment of hERG blockers with previous experimental or clinical annotation among the five enriched clusters identified in Figure **2B** were further quantified with the hypergeometric test, with resulting prediction statistics summarized in Table **2**. Quantification of the resulting predictive power in Table **3** suggests an overall accuracy of 82% based on drugs for which experimental or clinical annotation is available (e.g., excluding the grey ‘untested’ drugs in Figure 2B), which is consistent with a test of ‘good’ quality based on previously published metrics [30]. While the pairwise gene expression profile correlations (Pearson coefficients) within the hERG inhibitor-enriched clusters are significantly higher than the correlations between enriched cluster drugs and non-enriched cluster drugs (medians of 0.14 and 0.01, respectively, Wilcoxon rank-sum test p-value < 2.23x10^-308^) (Figure 3, A & B), the corresponding distributions of pairwise chemical similarities (Tanimoto coefficients) are statistically different (as judged by a Wilcoxon rank-sum test comparing the inter-cluster chemical similarity of the hERG inhibitor-enriched clusters versus their similarity to drugs in other clusters, p-value 1.1510x10^-288^) yet possess approximately equal medians (0.12 and 0.10, respectively) (Figure 3C). Thus, this analysis highlights correlations in drug-induced gene expression profiles that are not evident from chemical similarity alone. Intriguingly, we also noted that the MCF7 Astemizole-exemplar cluster includes Miconazole and Mefloquine, drugs which have been previously shown to inhibit hERG channels recombinantly expressed in cell lines [31,32], but did not appear in our lists of clinically annotated LQT-causing drugs and were inactive in our high-throughput electrophysiology assay. This suggests that this dataset may contain false negatives which nevertheless cluster with other known inhibitors based on similarity in transcriptional responses. Conversely, compensatory block of other ionic currents in addition to hERG may normalize hERG effects by these drugs, leading to no observable clinical phenotype [33,34]. Examination of the relationship between a drug’s hERG inhibition and maximal Pearson correlation to any of the five hERG blocker enriched cluster exemplars in Figure **2B** demonstrates a modest linear correlation which is statistically significant compared to randomized data (Figure **S4, A & B**). Further, a greater fraction of drugs with high hERG inhibition are present in the enriched clusters of Figure **2B** than those with low hERG inhibition (Figure **S4C**). Evaluation of enriched gene ontology (GO) annotations among genes up and down-regulated in the five hERG inhibitor-enriched clusters indicated positive effects on *cholesterol biosynthesis* (GO:0006695), *isoprenoid biosynthesis* (GO:0008299), and the *unfolded protein response* (GO:0030968), and negative effects on *cell cycle checkpoint* (GO:0000075), *Sphase* *of mitotic cell cycle* (GO:0000084), and *DNA replication* (GO:0006260). The physiological correlation between hERG block and these processes remains to be investigated though intriguingly, previous reports have linked hERG channel activities to a variety of biological processes in addition to cardiac function [23,24,35,36]. Functional enrichment results for all clusters are given in Table **S3**.

**Table 2:** Statistical enrichment of hERG inhibitors in transcriptionally determined clusters.

Dataset	Drugs	Fraction of Tested (p-value hypergeometric test)
All Clusters	602 tested	
**Experimental Inhibitors**	62	0.10 (-)
**Annotated Inhibitors**	57	0.09 (-)
**Total Inhibitors**	119	0.20 (-)
Enriched Clusters	80 tested	
**Experimental Inhibitors**	27	0.34 (3.55e-11)
**Annotated Inhibitors**	19	0.24 (7.24e-6)
**Total Inhibitors**	**46**	0.58 (<3.33e-16)
Non-Enriched Clusters	522 tested	
**Experimental Inhibitors**	35	0.07 (2.65e-10)
**Annotated Inhibitors**	38	0.07 (3.11e-5)
**Total Inhibitors**	**73**	0.14 (3.55e-16)

**Table 3:** Prediction statistics for transcriptional-signature based hERG inhibitor enrichment.

Statistic	Value
**Sensitivity TP/(TP+FN)**	46/(46+73) = 39%
**Specificity TN/(TN+FP)**	449/(449+34) = 93%
**Overall Accuracy (Predictivity) (TP+TN)/(TP+TN+FP+FN)**	(46+449)/(46+449+34+73) = 82%

Data are derived from values for True Positive (TP) (46 drugs), True Negative (TN) (449 drugs), False Positive (FP) (34 drugs), and False Negative (FN) (73 drugs) in Table **2**.

### Experimental validation of predicted hERG inhibitors

**Figure 4:**
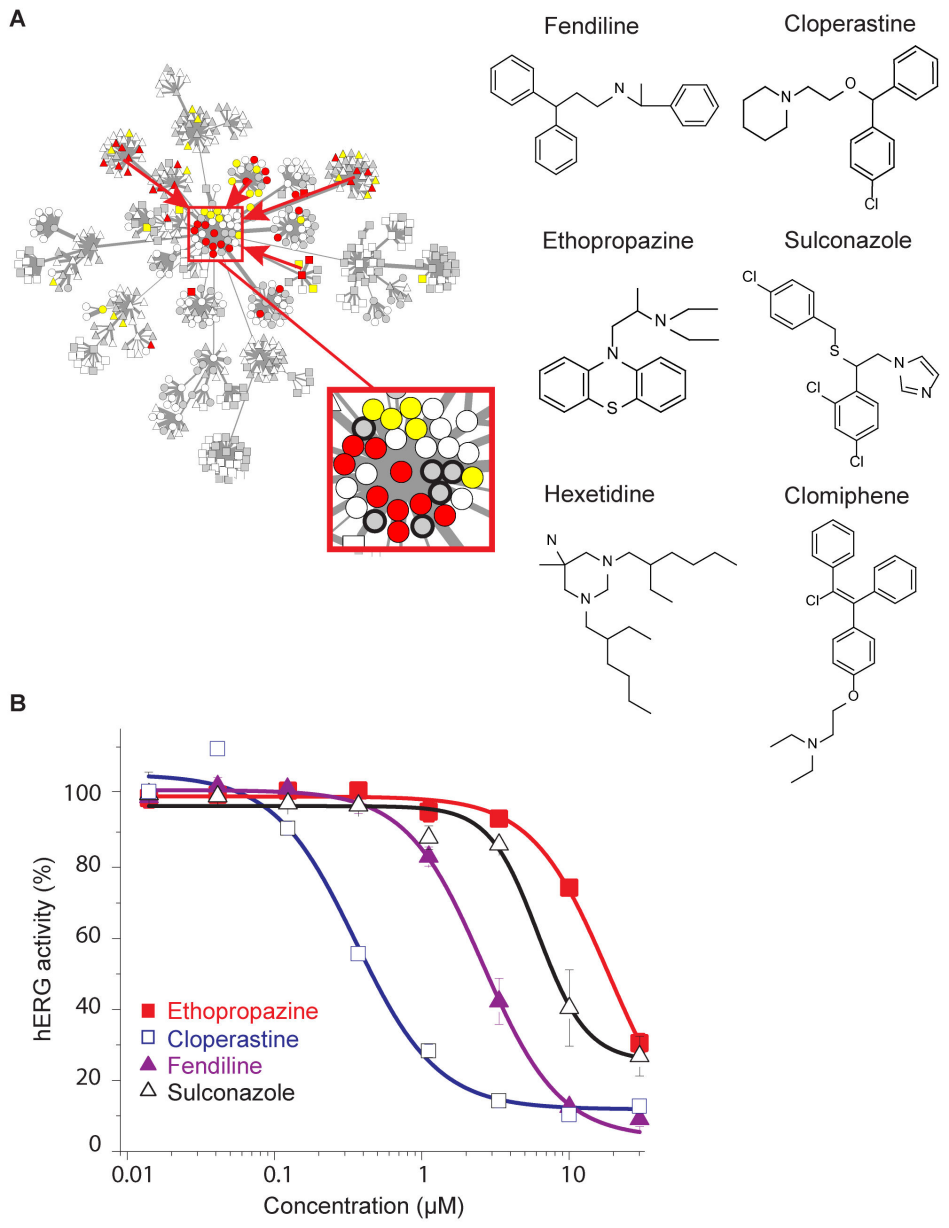
Experimental validation of novel hERG inhibitors. (**A**) (Left) Exemplars of hERG inhibitor enriched clusters from Figure **2B** converge at the MCF7-derived Astemizole cluster (red arrows, inset), which contains six unannotated drugs (black highlights in inset) (Right). Chemical structures of the six unannotated drugs in the highlighted cluster. (**B**) Dose response curves for hERG inhibition measured for four unannotated drugs using the Ionworks automated patch clamp system (n = 4, mean +/- s.e.m. for each data point).

To determine whether our analysis could predict novel hERG ligands among the compounds in the inhibitor-enriched clusters, we examined drugs without existing experimental or clinical annotation from the databases used in our analysis in these groups. Figure **4A** demonstrates that the Astemizole cluster from the MCF7-tested drug set is the overall center of all hERG inhibitor enriched clusters, representing the most characteristic pattern for these five groups. The structures of the six ‘missing-data’ drugs in the MCF7 Astemizole cluster (Figure 4A) display limited structural similarities, and differences in functional moieties (such as the three chloro-groups of Sulconazole) that could reasonably alter their surface polarities, along with variation in linker group composition (with Fendiline, Sulconazole, and Cloperastine bearing, respectively, a nitrogen, sulfur, and oxygen atom along their carbon backbones). Four of these compounds (Fendiline, Cloperastine, Ethopropazine, and Sulconazole) were untested in our previous electrophysiology data (http://www.hERGcentral.org) [26] and lacked annotation for drug-induced LQT syndrome (http://ww.sads.org.uk and http://www.qtdrugs.org). Though not included in the LQT drug lists we utilized, we found previous literature associating Fendilene with drug-induced LQT [37], but no evidence of its direct inhibition of hERG current. Additionally, Sulconazole has been previously annotated in the bioactivity records in the ChEMBL database as inactive in a hERG binding assay using Astemizole displacement as a functional readout [38]. The remaining compound in the Astemizole cluster, Clomiphene, was not in the databases used to annotate Figure **2B** but has been previously shown to inhibit the current of recombinantly expressed hERG channels with an IC_50_ of 0.18 µM [39]. We could locate no previous data describing Hexetidine, Cloperastine, or Ethopropazine effect on hERG or association with LQT. We therefore evaluated several of these unannotated compounds experimentally for inhibition of hERG current using automated electrophysiology recordings of a Chinese hamster ovary (CHO) stable cell line, a popular expression system due to its low background current from endogenous potassium channels compared to human epithelial kidney 293 (HEK293) cells [40,41]. With the exception of Hexetidine, which appears to disrupt the membrane seal during recording, the remaining four were successfully tested. Figure **4B** shows the measured dose–response curves, for which we calculated IC_50_ values of 18.6 ± 0.8 µM (Ethopropazine, n = 4), 0.36 ± 0.05 µM (Cloperastine, n = 4), 2.6 ± 0.1 µM (Fendiline, n = 4), and 6.1 ± 0.9 µM (Sulconazole, n = 4). Because the Ionworks platform used in our assessment tends to underestimate the potency of compounds [42,43], the effective concentrations could be even lower.

To assess the statistical significance of the results, we simulated 1,000 random sets of four compounds using the distribution of previously recorded experimental data for 10 µM inhibition of hERG current for the subset of MCF7-tested drugs (those present in the network of Figure 2A) in our high-throughput electrophysiology assay (Figure **S5A**), finding that the average percent inhibition of the tested compounds (67%, or a hERG activity of 33%) was greater than any random group (an empirical p-value of <0.001) (Figure **S5B**). Therefore, our clustering analysis significantly enriches for hERG inhibition among previously unannotated compounds.

## Discussion

In this study we identified commonalities in the transcriptional responses of structurally diverse hERG inhibitors, suggesting microarrays as a novel proxy measurement correlated with conduction of potassium currents by hERG and hence liability of channel block. Perhaps as remarkably, the observed change in gene expression is not necessarily a direct result of hERG inhibition. While hERG inhibitor-enriched clusters were observed for profiles generated in all three cell lines, functional evidence for hERG expression has been reported only for MCF7 and HL60 [23,24], indicating that channel expression may not be strictly required for the observed pattern. Indeed, there are also reports that channels including hERG have activities in addition to ion conductance [44–46], indicating that independent molecular targets might converge on common signaling pathways or processes also modulated by hERG and leading to the observed correlation. For example, there is a tendency that hERG inhibitors or LQT-causing drugs are also antagonists of the multidrug resistance transporter (MDR) [47]. Alternatively, hERG may be co-expressed with other channels correlated with oncogenesis which possess similar pharmacological profiles, such as hEAG [48], thus confounding causal inference of the relationship between hERG activity and gene expression response.

We also note that the presence of inhibitors in the CMap outside the enriched clusters highlighted in our analysis indicates that this “hERG signature” is not necessarily “dominant” over other expression pattern(s), implying that other such patterns might perturb or mask the signature from being identified for some compounds. In this interpretation, the subset of clustered inhibitors highlighted in our analysis represent drugs for which this signature is dominant over or of equal strength with other expression responses of the compounds. Additionally, we note that a large portion of the compounds in this dataset exhibit silent or weak transcriptional response which prevents profiling for hERG inhibition using the proposed approach. As the signatures in CMap are uniformly generated at a 6 hour time point, it is possible that some compounds may display chronic transcriptional effects at a later time point, and thus be profiled by modifications in the original screening protocol. Indeed, previous studies of time course data from drug-induced gene expression responses have indicated that distinct expression patterns may be detected at different time points [49–51], even for frequent measurements such as 3, 6, and 9 hours. We thus hypothesize that some of the ‘silent compounds’ in our study might have detectable signatures at later time points, while the hERG inhibitors outside of enriched clusters may exhibit a dominant ‘hERG signature’ at earlier time points. Taken together, these results suggest that improved sensitivity for this assay might be achieved by using time course instead of single point expression data. Additionally, we note that the sensitivity of our assay may be effected by our choice of a 10 µM IC_50_ threshold. While this threshold has been used in previous hERG predictive models [52], previous studies have also reported greater accuracy with a lower threshold (e.g., IC_50_ 40 µM) [53]. Thus it may also be possible to improve the sensitivity of our method using inhibition measurements derived from higher drug concentrations.

**Figure 5:**
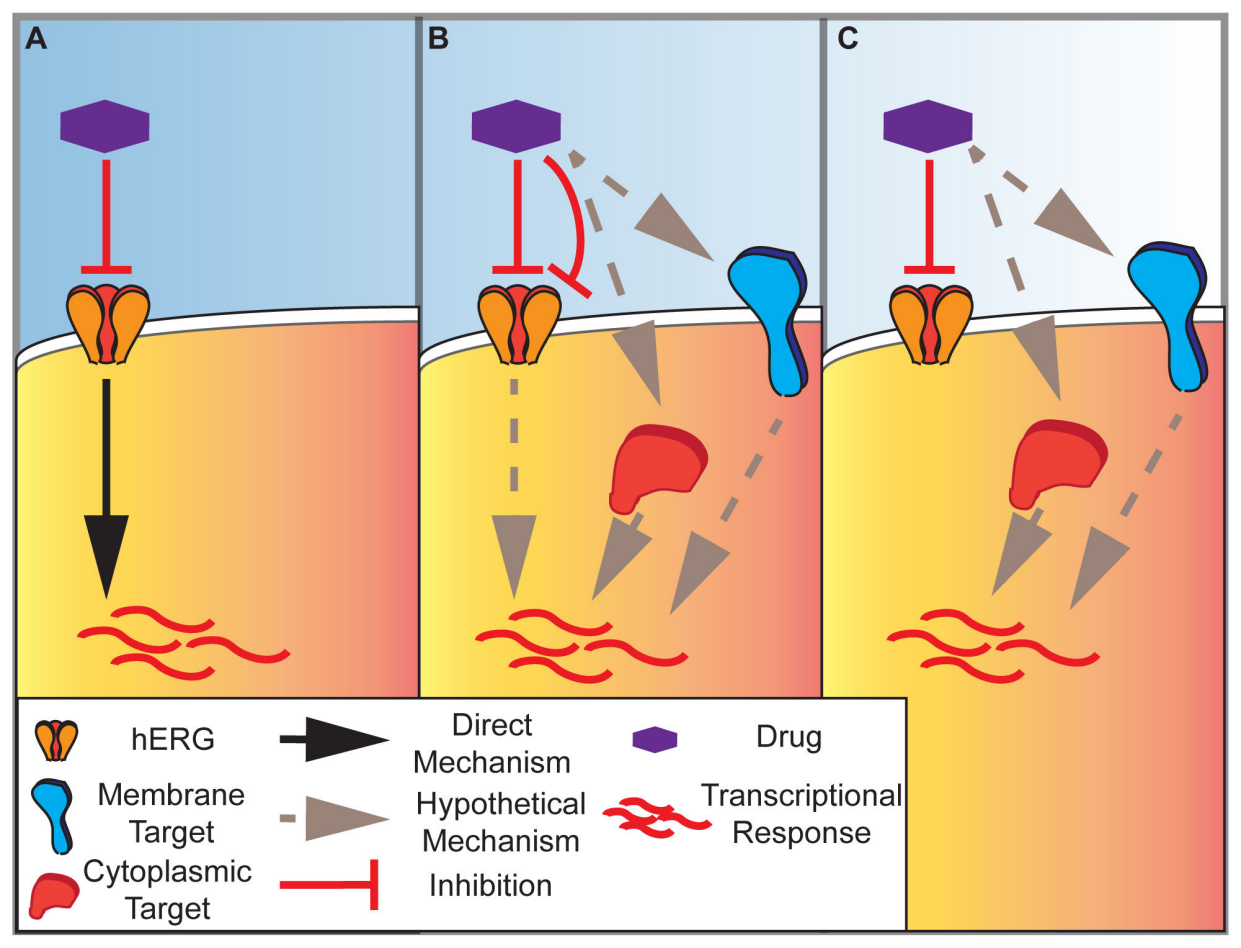
Mechanistic hypotheses for hERG-inhibition correlated gene expression signatures. (**A**) Schematic of drug-induced gene expression response directly controlled by blockade of potassium conductance by the hERG channel. (**B**) Parallel direct (straight repression line) or indirect (bent repression line) modulation of hERG and alternative molecular targets on the cell membrane (blue) or in the cytoplasm (red) may lead to convergent transcriptional responses. (**C**) Perfect confounding, in which drugs simultaneously inhibit channel function and independently modulate downstream transcriptional response through alternative molecular targets.

Research has also suggested that the duration of action potentials at 90% repolarization (APD90), a correlate of clinical LQT which is elongated by hERG inhibition, may be dependent upon multi-channel drugs effects [33,34], and thus the ability of our approach to forecast clinical endpoints may be aided by future integration of high-throughput recording data for other cardiac channels such as Nav1.5. Furthermore, despite the current lack of causal evidence linking the gene-expression profiles of the clustered hERG inhibitors in the CMap with functional modulation of the channel, this analysis does suggest an intriguing possibility that some hERG inhibitors induce a downstream signaling cascade as a consequence of current reduction that is visible as a global change in gene expression. Alternatively, these observations may indicate signaling pathways downstream of potassium channels that are not directly related to their role in conduction [44]. A selection of these hypotheses is diagrammed in Figure **5**. Certainly, profiling selective inhibitors of hERG such as E4031 which are not present in the CMap might help clarify these hypotheses, though the large number of transcriptionally silent compounds in the dataset suggests these selective inhibitors may not exhibit a detectable signature at 6 hour time points.

From a practical standpoint, the observed similarity of microarray profiles among electrophysiologically confirmed but structurally diverse inhibitors argues for the potential of using such a surrogate as an informative descriptor for hERG liability complementing existing electrophysiological assays. The utility of such a platform is suggested by compounds such as the antidepressant Amoxapine [54] which display slow on-rates beyond the temporal resolution of high throughput electrophysiological systems (which is typically less than 5 minutes), and thus appear as ‘negatives’ in our acute experimental electrophysiology data. In contrast, the microarray data utilized in this study were generated 6 hours following drug treatment, suggesting gene expression measurements may offer complementary temporal resolution not readily accessible by automated electrophysiology data, allowing high-throughput assessment of hERG inhibition in compounds with slow on-rates which have previously required manual patch clamp recordings to resolve. Furthermore, transcriptional signatures may identify false negatives from other assays, such as Sulconazole, which was labeled as inactive in ChemblDB from binding data. Because binding experiments often utilize displacement of a known ligand, they will not identify compounds binding at alternative sites(s) of action.

Gene expression measurements have additional attractive properties compared to other high-throughput technologies. Because microarray profiles represent an integrated output of multiple signaling pathways in the cell, they are potentially more sensitive than biochemical or cellular assays which are commonly designed to test one or a limited number of physiological parameters. Such expression profiles are also certainly more general in terms of measuring diverse signaling pathways and integrated biological events. Thus, assessment of hERG liability may be effectively evaluated in parallel with other endpoints of biological interest, such as inflammatory signaling, oxidative damage response, or metabolic perturbations. Additionally, the fact that our signature utilizes measurements in cancer cells derived from different tissues of origin suggests the attractive possibility of assaying the effects of hERG activity in these oncogenesis models, as previous research has linked hERG expression to tumor migration and cell volume [24,55]. Admittedly, cells with cardiac lineage may be equally or more informative. Indeed, patient-derived induced pluripotent stem cell (iPSC) models of cardiac disease have proven to be attractive disease models in electrophysiology studies [17,18,56], with additional evidence suggesting the potential for cardiac-specific transcriptional activity that may find utility in genomic drug-activity profiles [57–59]. Combined with cost savings generated by custom arrays that measure only the subset of differentially expressed genes correlated with hERG risk, these aspects suggest the potential for a novel genetic platform to assess ion channel activity.

More generally, our analysis contributes to growing evidence that systems-level measurements of drug effect reveal connections and similarities often invisible from the perspective of single molecular descriptors or activity measurements [6,9,18,60,61]. These links suggest not only the possibility of mining such connections for predictive purposes, but also that the full pharmacological complexity of even long-standing medications may not yet be appreciated. Integrated analyses are thus poised to illuminate these patterns and suggest possibly novel indications or, as in our study, liabilities of existing drugs.

## Methods

### Gene expression and drug property/activity data

All raw data comprising the Connectivity Map (CMap) build 02 were downloaded as CEL files from the Broad Institute (http://www.broadinstitute.org/cmap/). We annotated compounds for percentage of hERG inhibition using data from a previously described database of high-throughput electrophysiology measurements [26], and obtained LQT-risk data from online references at www.sads.org.uk and www.qtdrugs.org. Simplified molecular input line entry system (SMILES) strings representing the chemical structures of all compounds were downloaded from PubChem and ChemBank. Computational filtering of salts, standardization of charge and coordinates, and calculation of functional circular fingerprints (FCFP_6) were performed with Pipeline Pilot Student Edition v 6.1 (Scitegic).

### Microarray pre-processing

Our analysis of the Connectivity Map Build 02 data consisted of four steps:

#### Platform selection and probeset normalization

From the 7,056 cell files in the CMap build 02, we selected 6,029 files generated from HT-HG-U133A arrays, consisting of 5,242 drug treatment instances and 967 DMSO-treated vehicle controls tested on three tumor cell lines of human origin. This selection is performed because unlike the non-parametric processing used in the original analysis [9], the probeset normalization algorithms we employed require a homogenous platform. All selected CEL files were probeset-normalized using GC robust multi-array average (GCRMA) background correction as implemented in the *aroma. affymetrix* R package [62,63] in R 2.14.2 [64].

#### Batch correction

Following pre-processing, we sought to remove correlations between arrays due to experimental batch (date of data acquisition) rather than biological similarity by mean-centering probesets across all drugs in each batch, following a previously described pipeline [27]. Since this correction assumes that on average a probeset should not be differentially expressed among an experimental batch of otherwise unrelated drugs, we retained only batches with sufficient numbers (>25) for this assumption to reasonably hold. To provide the most consistent comparison between replicates of the same drug in the same cell background, we retained only arrays representing the single concentration with the most examples for a given drug (the mode). However, we also note that the variation of test concentrations across CMap instances for a given drug is not often large, and thus this source of variability is likely minor. Applying these two criteria left 4,754 drug treatment and 700 control instances.

#### Filtering Transcriptionally Inactive Drugs

It has previously been reported that some drugs in the CMap are “inactive”, as judged by lack of correlation among replicates (arrays representing transcriptional response to the same drug in the same cell background at the same concentration), but only pairs of drugs tested more than once were considered in previous analysis [65]. We sought a universal filter to apply to all the data to filter these “transcriptionally silent” drugs. In order to filter these “transcriptionally silent” treatment profiles, drug treatment instances without at least 10 probesets exceeding 2 log_2_ fold units expression change (increase or decrease) compared to batch mean (representing vehicle treated control) and at least 1 probeset exceeding 3 log_2_ fold units expression change compared to batch mean (increase) were removed, a heuristic criterion that generated the bimodal distribution displayed in Figure **S1**. This yielded a final set of 1,419 drug treatment arrays representing 673 unique compounds tested on three cancer cells lines. Probeset values for replicate measurements of a given drug-cell line pair were averaged, yielding 1,033 unique combinations of drug and cell background.

#### Identifying Differentially Expressed Genes

Differentially expressed probesets were determined by calculating the 2.5 and 97.5 percentiles of log_2_ expression ranges for a given probeset among the DMSO-treated vehicle controls for a given cell line, and setting to 0 all probeset values in drug treatment instances that were not outside this range.

### Clustering and enrichment analysis

Statistical analysis of the resulting drug-induced gene expression profiles was performed in MATLAB R2012a (The Mathworks). The similarity between drug treatment profiles in each cell line were quantified using Pearson correlation, and clustered using affinity propagation, a message-passing algorithm that automatically splits datasets into clusters defined by a set of distinct exemplars (centroids) [28], with the probability of each drug becoming a cluster exemplar being set to the median of pairwise Pearson correlations. To aggregate the data further, the exemplars identified in the initial clustering were also grouped using affinity propagation (with the initial probability of being a cluster exemplar held to the original group median used in the first round of clustering), and this process was repeated hierarchically until the resulting clusters could not be further merged, in a manner similar to previous analysis of the CMap [6]. Chemical similarity between compounds was computed using the Tanimoto coefficient (Jaccard coefficient) using FCFP_6 circular fingerprints calculated in Pipeline Pilot (Scitegic).

To calculate cluster enrichment for hERG inhibitors (>50% reduction of activity at 10 µM or LQT side effect) through permutation testing, labels among compounds experimentally or clinically annotated in our databases were randomized 1000 times and the number of times the resulting cluster enrichments (fraction of hERG inhibitors or LQT drugs among all annotated compounds in a cluster) exceeded the observed number of hERG inhibitors and LQT drugs in the clusters in Figure **2B** was computed. This count yielded an empirical p-value (number of times out of 1000 permutations that the enrichment of a randomized cluster exceeded the observed enrichment in the clusters of Figure 2B) which was adjusted using the Benjamini–Hochberg procedure [60] to control for multiple hypothesis testing employing a false discovery rate of 0.2. Clusters were visualized using Cytoscape 2.8.2. Gene Ontology (GO) analysis was performed using the topGO package in bioconductor [66], using the Fisher’s exact test and the *elim* method for the Biological Process ontology. For each cluster, the set of genes that were up or down-regulated in at least half of the cluster members (median greater or less than 0) were tested for GO term enrichment.

### Experimental validation of hERG inhibitors

Ethopropazine, Cloperastine, Fendiline, and Sulconazole (Sigma Aldrich) were prepared at 30 µM stock concentration and serially diluted 3-fold for eight-point dose response measurements. Inhibition of hERG current was experimentally assessed using a previously described protocol [19]. Briefly, Chinese hamster ovary (CHO) cells stably expressing the hERG channel were dislodged from tissue culture flasks and dispensed into PPC plates. Background leak currents were estimated by initiating a 100 ms step to -80 mV from an initial holding potential of -70 mV and subtracted from the subsequent current measurement. For each drug concentration, sequential voltage pulses were applied, each using a 100 ms step to -30 mV from a holding potential of -70 mV, a 2 s conditioning step to +45 mV, and a 2 s test step to -30 mV. Small molecule effects on hERG current density were quantified by measuring the peak tail current prior to compound application and dividing by the amplitude following application of each test concentration. Recordings with peak tail current amplitude pre-compound > 0.2 nA, seal resistance > 30 MOhms, and seal resistance drop rate < 25% were retained for subsequent analysis. Data were fit to a sigmoidal dose response curve using Origin 6.0 (Microcal). To assess the statistical enrichment of this result, we simulated 1000 random sets of four compounds drawn from our experimental data for 10 µM hERG inhibition for the MCF7-tested drugs (red or white nodes in Figure 2A) and compared the mean inhibition of these random sets to that observed for the tested compounds.

## Supporting Information

Figure S1 Distribution of pairwise correlations among transcriptionally active and silent drugs. Replicate drug treatments (duplicate microarray instances for the same concentration, cell line, and drug) may be divided into populations of transcriptionally active (red) and silent (black) drugs based on filters for the magnitude of log_2_ fold expression change compared to batch average (representing vehicle treated control) among probesets of a given drug.
(TIF)

Figure S2 LQT-annotated drugs have statistically enriched hERG inhibition. Boxplot of distributional difference in experimentally recorded hERG inhibition values for LQT annotated and unannotated drugs.
(TIF)

Figure S3 Cell line dependent and independent expression changes in hERG-inhibitor enriched clusters. (**A**) X, Y, and Z (color gradient of scatterplot points) axes denote average log_2_ fold gene expression changes versus DMSO treated controls for drugs in hERG-inhibitor enriched clusters highlighted in Figure **2** for drugs profiled in PC3 (x axis) HL60 (y axis) and MCF7 cells (z axis, color gradient of scatterplot points). Purple, red, and blue dashed boxes denote regions of cell-line specific transcriptional modulation. Red or blue shaded points within orange dashed box denote genes with cell line-independent transcriptional changes upon drug treatment. (**B**) Overlap of differentially expressed (DE) genes with average change in expression greater than 0 versus DMSO treated controls for drugs in the hERG-inhibitor-enriched clusters highlighted in Figure **2B** profiled in the three cancer cell lines utilized in the CMap. (**C**) As in (**B**), for genes with average fold change less than 0 in the highlighted clusters.
(TIF)

Figure S4 Correlation between hERG activity and transcriptional similarity. (**A**) Measured hERG activity (%) is plotted for all assayed drugs versus the maximum correlation (Pearson coefficient) judged by gene expression microarray to one of the five drug expression profiles at the centers of the five enriched cluster exemplars in Figure **2B**. (**B**) Comparison of the correlation in (**A**) to that in 1,000 sets in which drug activities have been randomly permuted. (**C**) Fraction of drugs in enriched clusters (red) in Figure **2B** for all drugs within a given range (window of activity values with width 10) of measured hERG activity (%).
(TIF)

Figure S5 Statistical evaluation of hERG inhibition among validated compounds. (**A**) Distribution of experimentally measured hERG inhibition for all compounds tested on the MCF7 cell line in Figure **2A**. (**B**) Mean hERG activity of random sets of 4 drugs selected from the distribution of (**A**), compared to the set of validated inhibitors in Figure **4**.
(TIF)

Table S1 Drug annotations, SMILES strings. (XLSX)

Table S2 Drug network for MCF7, all cells. (XLSX)

Table S3 GO annotations for drug network clusters. (XLSX)
