# Supplementary figures and images for: Integrated Analysis of Drug-Induced Gene Expression Profiles Predicts Novel hERG Inhibitors
Source: PLoS One. 2013 Jul 23;8(7):e69513. doi: 10.1371/journal.pone.0069513 (PMC3720659; doi:10.1371/journal.pone.0069513)

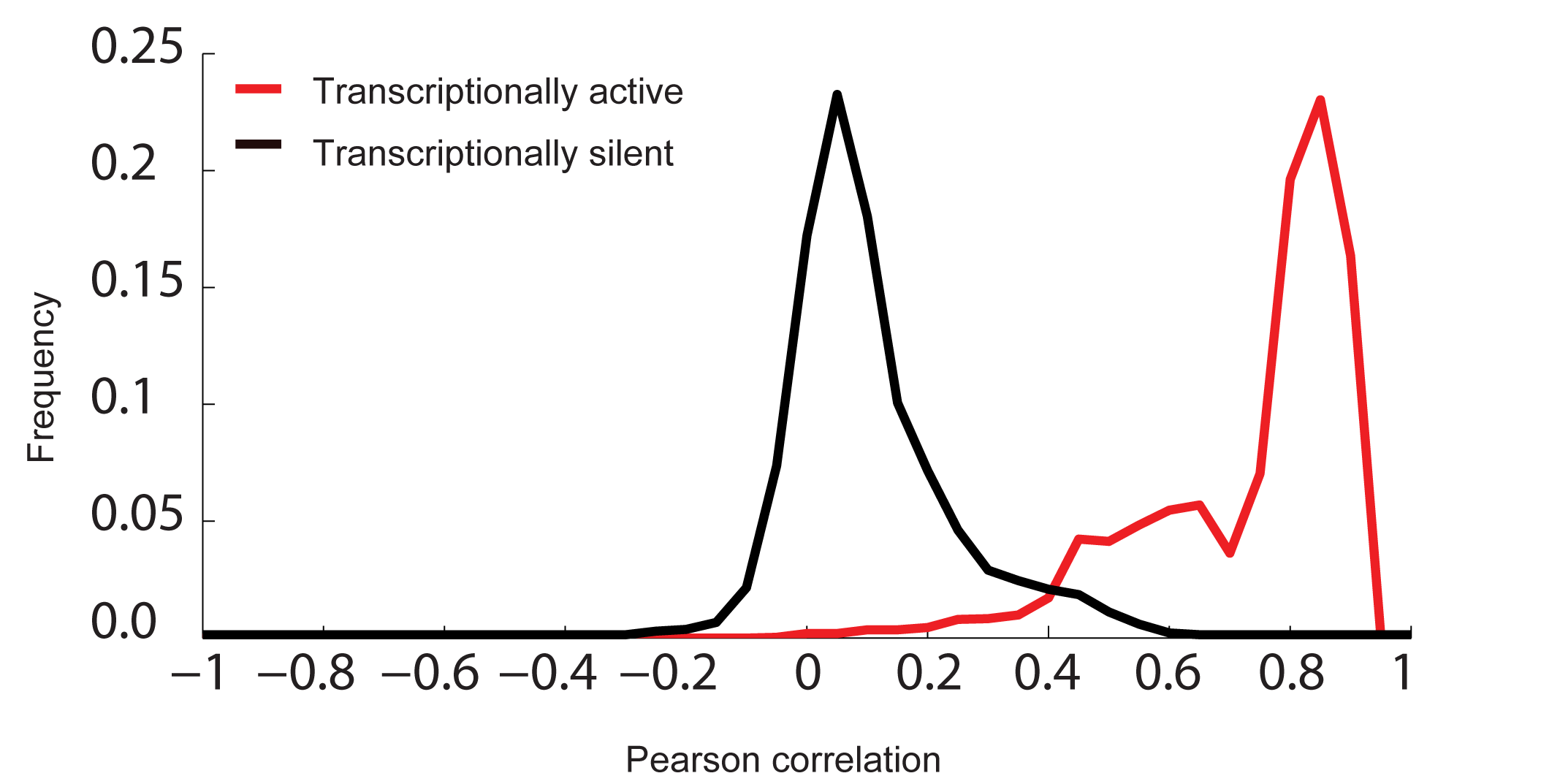

Supplement: Figure S1 — Replicate drug treatments (duplicate microarray instances for the same concentration, cell line, and drug) may be divided into populations of transcriptionally active (red) and silent (black) drugs based on filters for the magnitude of log2 fold expression change compared to batch average (representing vehicle treated control) among probesets of a given drug. (TIF) [file pone.0069513.s001.tif]

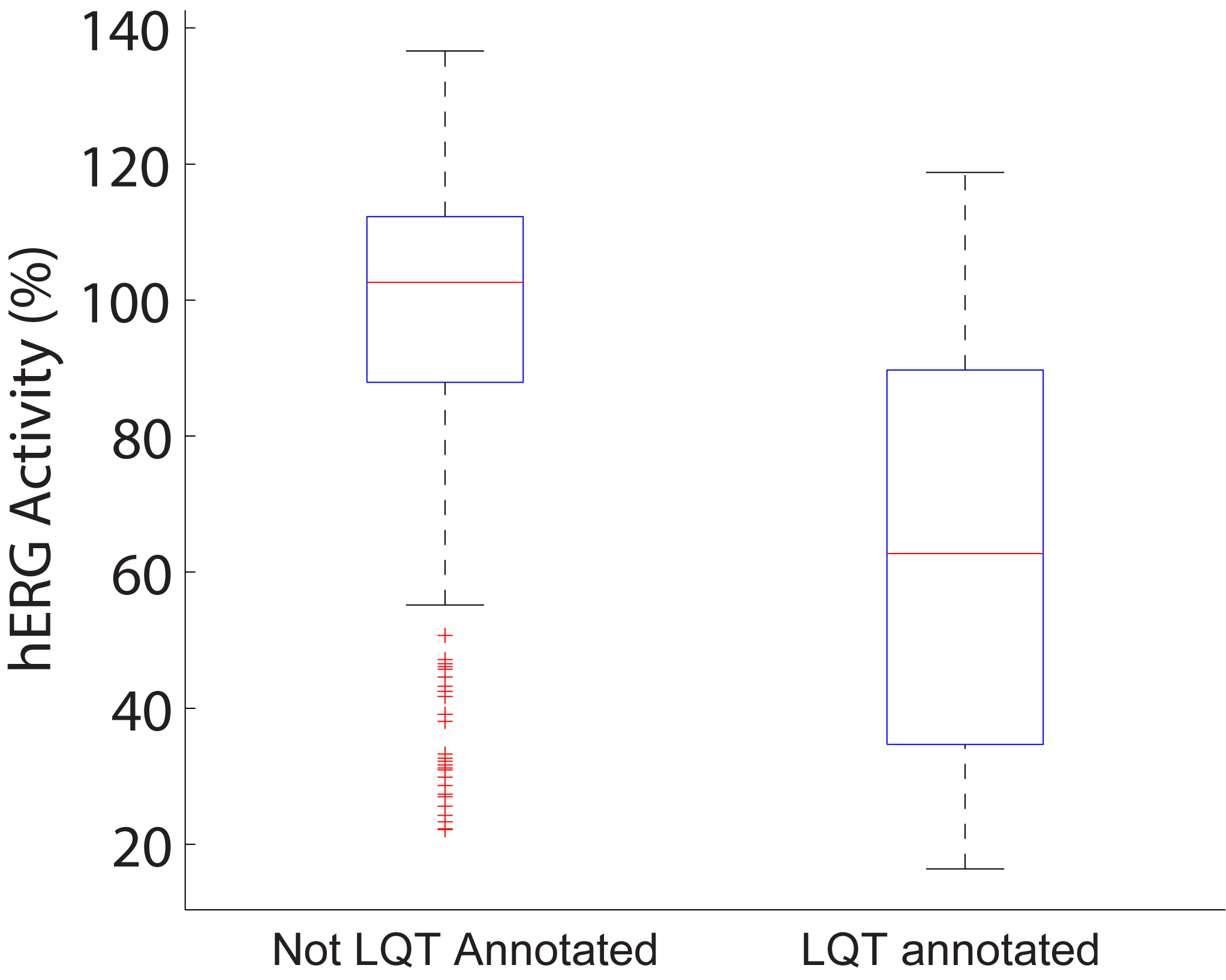

Supplement: Figure S2 — Boxplot of distributional difference in experimentally recorded hERG inhibition values for LQT annotated and unannotated drugs. (TIF) [file pone.0069513.s002.tif]

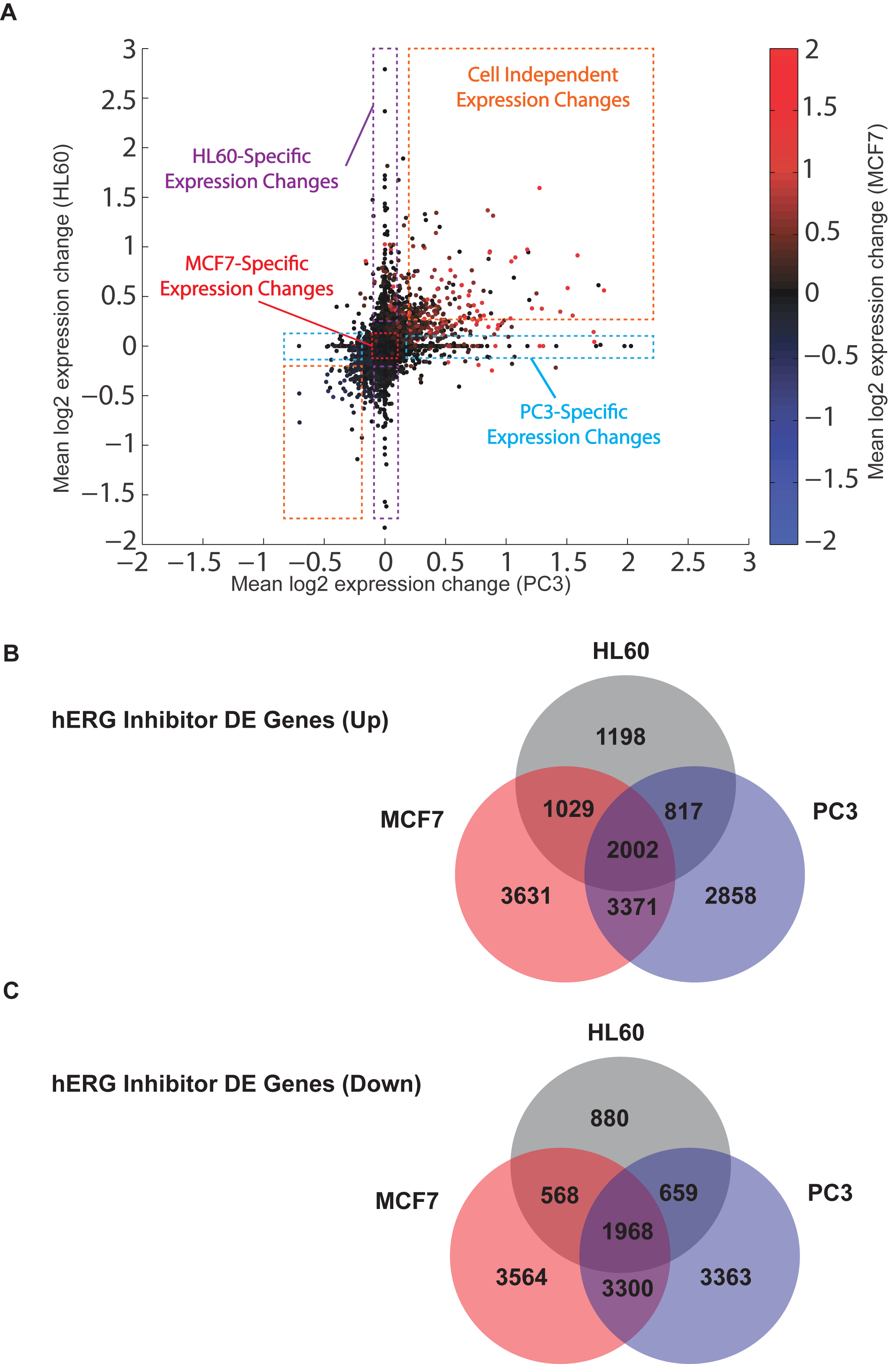

Supplement: Figure S3 — (A) X, Y, and Z (color gradient of scatterplot points) axes denote average log2 fold gene expression changes versus DMSO treated controls for drugs in hERG-inhibitor enriched clusters highlighted in Figure 2 for drugs profiled in PC3 (x axis) HL60 (y axis) and MCF7 cells (z axis, color gradient of scatterplot points). Purple, red, and blue dashed boxes denote regions of cell-line specific transcriptional modulation. Red or blue shaded points within orange dashed box denote genes with cell line-independent transcriptional changes upon drug treatment. (B) Overlap of differentially expressed (DE) genes with average change in expression greater than 0 versus DMSO treated controls for drugs in the hERG-inhibitor-enriched clusters highlighted in Figure 2B profiled in the three cancer cell lines utilized in the CMap. (C) As in (B), for genes with average fold change less than 0 in the highlighted clusters. (TIF) [file pone.0069513.s003.tif]

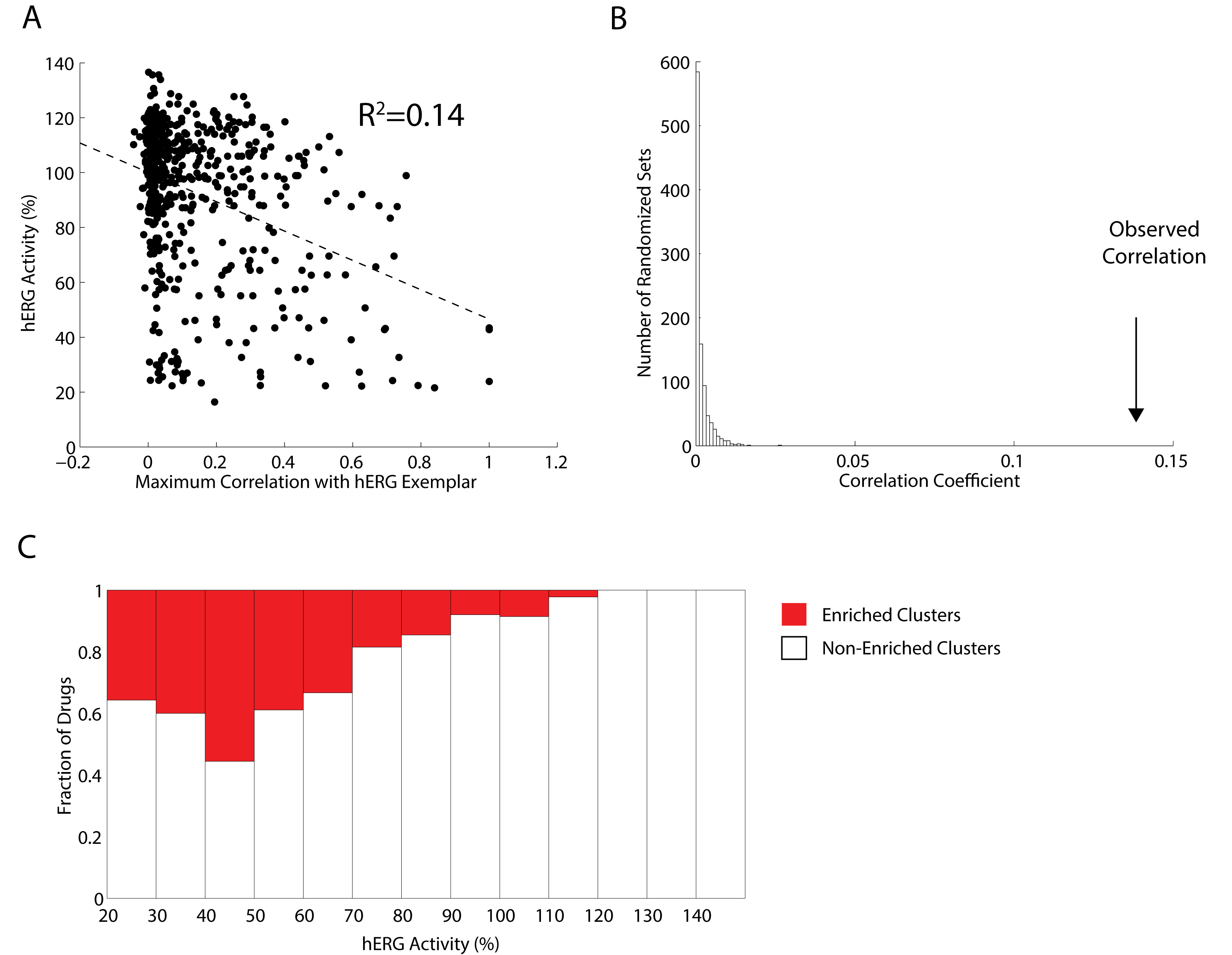

Supplement: Figure S4 — (A) Measured hERG activity (%) is plotted for all assayed drugs versus the maximum correlation (Pearson coefficient) judged by gene expression microarray to one of the five drug expression profiles at the centers of the five enriched cluster exemplars in Figure 2B . (B) Comparison of the correlation in (A) to that in 1,000 sets in which drug activities have been randomly permuted. (C) Fraction of drugs in enriched clusters (red) in Figure 2B for all drugs within a given range (window of activity values with width 10) of measured hERG activity (%). (TIF) [file pone.0069513.s004.tif]

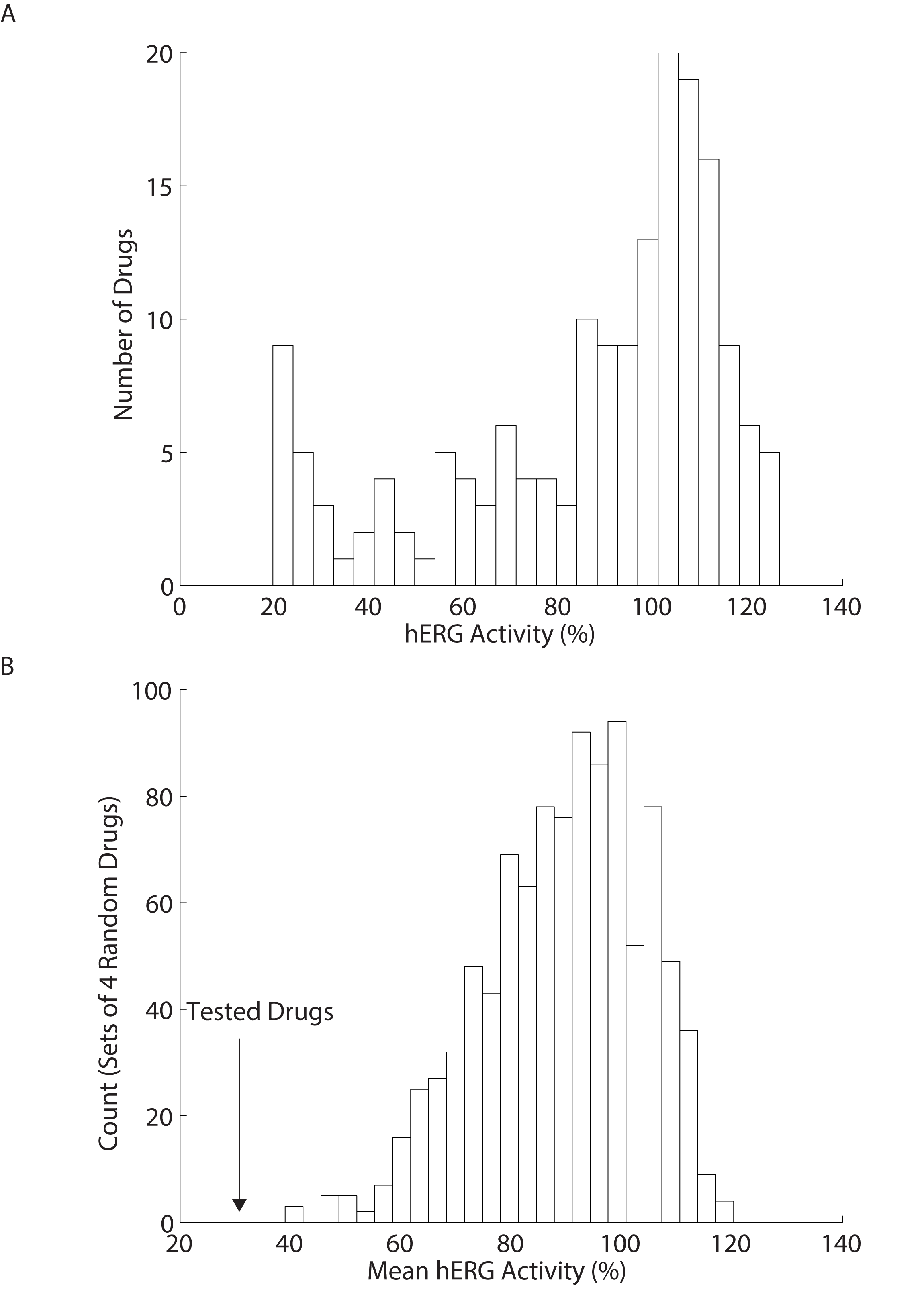

Supplement: Figure S5 — (A) Distribution of experimentally measured hERG inhibition for all compounds tested on the MCF7 cell line in Figure 2A . (B) Mean hERG activity of random sets of 4 drugs selected from the distribution of (A), compared to the set of validated inhibitors in Figure 4 . (TIF) [file pone.0069513.s005.tif]
